# Evaluating the Safety and Outcomes of Oesophagogastroduodenoscopy in Elderly Patients Presenting With Acute Upper Gastrointestinal Bleeding

**DOI:** 10.7759/cureus.47116

**Published:** 2023-10-16

**Authors:** Alexandra McWhirter, Saba Mahmood, Ekow Mensah, Hussameldin M Nour, Olaolu Olabintan, Ziva Mrevlje

**Affiliations:** 1 General Internal Medicine, Royal Sussex County Hospital, University Hospitals Sussex NHS Foundation Trust, Brighton, GBR; 2 Geriatrics, Royal Sussex County Hospital, University Hospitals Sussex NHS Foundation Trust, Brighton, GBR; 3 General Surgery, Furness General Hospital, University Hospitals of Morecambe Bay NHS Foundation Trust, Brighton, GBR; 4 Gastroenterology, King's College Hospital NHS Foundation Trust, London, GBR; 5 Gastroenterology, Royal Sussex County Hospital, University Hospitals Sussex NHS Foundation Trust, Brighton, GBR

**Keywords:** upper gastrointestinal bleed, oesophagogastroduodenoscopy (ogd), emergency gastroenterology and endoscopy, clinical frailty score, co-morbidities, elderly population

## Abstract

Aims: In the absence of evidence-based guidelines regarding the safety and appropriateness of emergency endoscopy in elderly, co-morbid and frail patients, we aimed to find clinical outcomes in elderly patients who have undergone gastroscopy following an acute upper gastrointestinal bleeding (UGIB).

Methods: We carried out a retrospective observational study of patients aged 70 years and older who had undergone emergency oesophagogastroduodenoscopy (OGD) at the Royal Sussex County Hospital, Brighton, United Kingdom, between May 2020 and January 2022. Data collected for analysis included Glasgow-Blatchford score, age, gender, endoscopic findings, endoscopic treatments, immediate complications, 90-day complications, 30-day and 90-day survival, length of hospital stay and re-bleeding.

Results: A total of 248 study participants were categorised into two groups: age 70-79 years (n=102) and ≥80 years (n=146). Melaena (n=226, 91%, p=0.0001) was the commonest indication for emergency OGD in both groups, with the majority of patients presenting with a Glasgow-Blatchford score of ≥1 (n=200, 80.6%, p=0.2). Endoscopy findings were normal in 26.4% (n=27) of those 70-79 years and 32% (n=47) of those ≥80 years (p=0.01). Duodenal ulcer, oesophagitis and gastric ulcer were the commonest abnormal findings (n=50, 20%; n=29, 11.7%; and n=28, 11.3%, respectively). Of the participants, 93.8% (n=212) had no immediate complications. Bleeding and hypotension occurred in 2.7% (n=6) and 2% (n=5) of patients, respectively. At 90 days post-procedure, 83.3% (n=85) of those 70-79 years and 67.8% (n=99) of those ≥80 years had survived (p=0.180).

Conclusions: We conclude that OGD is largely a safe procedure in older adults with acute UGIB; however, the high proportion of OGDs with normal findings reinforces the importance of careful selection of patients.

## Introduction

Upper gastrointestinal bleeding (UGIB) is defined as intraluminal haemorrhage proximal to the ligament of Treitz [1]. The clinical presentation of upper GI bleeding can include haematemesis, coffee ground vomiting and melaena, and depending on aetiology and severity can result in significant haemodynamic instability [2]. Upper GI bleeding is a common condition, with a UK incidence ranging from 84 to 172 per 100,000 of the population per year, and mortality remains around 10%, despite changes in medical management over the last 50 years [3]. There are multiple causes of upper GI bleeding, such as peptic ulcer disease (which is most common), Mallory-Weiss tears, varices, oesophagitis, malignancy, vascular malformations and idiopathic [3].

The initial management of UGIB involves resuscitation, including transfusion of blood products with restrictive transfusion thresholds, correction of coagulopathies and the use of additional medication based on the suspected aetiology of the bleed such as proton pump inhibitors (PPIs), terlipressin and intravenous antibiotics. The endoscopic management of peptic ulcer bleeding can include injection and thermal or mechanical treatment using clips. The management of oesophageal varices is band ligation, while the management of gastric varices is dependent on their location and continuity with any oesophageal varices and may include ligation or injection of tissue glue or thrombin [4].

The diagnosis and management of UGIB in elderly patients can be challenging. The prevalence of UGIB increases with age, with as many as 70% occurring in those over 60 years [5]. As life expectancy increases, we are presented with an ageing population who are more likely to suffer from frailty syndromes and co-morbidities that increase their vulnerability to complications from UGIB and its associated management [6]. As a result, the mortality rate in those over 60 years increases to 12%-35%, in comparison to the general population where it is 10% [5].

In the management of UGIB, there is an emphasis on the provision of endoscopy within 24 hours [6]; however, this comes with an associated risk of complications relative to routine endoscopy. These include both gastrointestinal (bleeding and perforation) and non-gastrointestinal risks (cerebrovascular event, myocardial infarction and congestive heart failure), which in the elderly are largely related to the presence of co-morbidities and sedation used during the procedure [7]. Although there are some studies that suggest that endoscopy is a safe and useful diagnostic and therapeutic tool in elderly patients, these often include those performed in the outpatient or non-emergent setting [8] or include a wide age range [9]. In this project, we have chosen to focus on patients who have undergone gastroscopy following acute UGIB, as there is currently no guideline on deciding suitability for gastroscopy based on patient age, co-morbidities and frailty in the elderly cohort. Due to the increased risks of this procedure, it is important that appropriate patients are selected to ensure that the proposed benefits far outweigh the risks.

This article was previously presented as a poster at the European Society of Gastrointestinal Endoscopy (ESGE) annual congress ESGE Days on April 20-22, 2023.

## Materials and methods

Study population

We carried out a retrospective observational study of patients who had undergone emergency oesophagogastroduodenoscopy (OGD) at the Royal Sussex County Hospital, Brighton, United Kingdom, between May 2020 and January 2022. All patients aged over 70 years who presented with upper GI bleeding symptoms (haematemesis or melaena) and who underwent emergency OGD were included in the study. In line with other studies [10], we separated patients into the very elderly cohort (aged over 80 years) and the elderly cohort (aged 70-79 years) for analysis.

Management of acute UGIB

At our hospital, patients presenting with acute upper GI bleeding received appropriate initial resuscitation such as the transfusion of blood products and reversal of coagulopathies, guided by the use of thromboelastometry blood tests (ROTEM). Empirical intravenous proton pump inhibitor or intravenous terlipressin and antibiotics were administered if the suspected aetiology of the bleed was peptic ulcer disease or varices, respectively. Where required, sedative agents used prior to endoscopy were IV midazolam and IV fentanyl. If deemed necessary, OGD took place under general anaesthetic in the operating theatre.

Data extraction

Patients’ medical and endoscopy records were used to collect data for analysis using a pre-agreed-upon extraction sheet. Data extracted included patient demographics, Glasgow-Blatchford score, endoscopic findings and endoscopic treatments. Furthermore, outcome data such as immediate complications, length of hospital stay, 30-day survival, 90-day survival, 90-day complications and re-bleeding were abstracted.

Statistical analysis

Data were summarised using descriptive statistics, and analysis was performed with Statistical Package for the Social Sciences (SPSS) version 28.0 (IBM SPSS Statistics, Armonk, NY). Categorical variables were compared using the chi-squared or Fisher exact test.

## Results

Patient demographics

The study included a total of 248 participants, with 102 patients aged 70-79 years and 146 patients aged ≥80 years. In both groups, males were overall more likely to present with acute UGIB than females. The difference, which was statistically significant (p=0.003), was much less pronounced in the older cohort, where 43.8% of patients were females (n=64), compared with 25% in patients aged 70-79 years (n=26) (Table 1).

**Table 1:** Descriptive statistics of demographics, indications and Glasgow-Blatchford scores

Parameters	Total (n=248) (number (%))	Age group = 70-79 (n=102)	Age group ≥ 80 (n=146)	p-value
Age (years) (mean)	81±6.7			
Sex				0.003
Female	90 (36.3)	26	64	
Male	158 (63.7)	76	82	
Indications				
Haematemesis	46 (18.5)	11	35	0.009
Melaena	226 (91.1)	102	124	0.000
Glasgow-Blatchford score				0.222
Low risk (0)	48 (19.4)	16	32	
High risk (>0)	200 (80.6)	86	114	

Clinical characteristics

The most common indication for emergency OGD in both groups was melaena (91.1%, n=226). However, a greater proportion of those aged ≥80 years presented with haematemesis (n=35, 24%) compared to those in the younger age group (n=11, 10%, p=0.009). The majority of patients presented with a Glasgow-Blatchford score of ≥1 (n=200, 80.6%, p=0.2), with no significant difference between groups (p=0.222) (Table 1).

Endoscopic findings and treatment

Endoscopic findings in both groups showed that 30% (n=74) were normal. Of the participants, 26.4% (n=27) were normal in those 70-79 years and 32% (n=47) were normal in those ≥80 years (p=0.01). Duodenal ulcers, oesophagitis and gastric ulcers were the commonest abnormal findings (n=50, 20%; n=29, 11.7%; and n=28, 11.3%, respectively). It was most common for patients who did not undergo any endoscopic treatment (n=185, 74.9%). The most common therapy employed at endoscopy was injection of adrenaline and clip (n=37, 15%), followed by clip (n=12, 4.8%) (Table 2).

**Table 2:** Comparison of OGD findings, treatment, complications, length of stay, re-admission and survival by age groups OGD: oesophagogastroduodenoscopy, GI: gastrointestinal, GAVE: gastric antral vascular ectasia, APC: argon plasma coagulation

Parameters	Total (n=248) (number (%))	Age group = 70-79 (n=102)	Age group ≥ 80 (n=148)	p-value
Endoscopic findings				0.01
Normal	74 (29.8)	27	47	
Duodenal ulcer	50 (20)	24	26	
Gastric ulcer	28 (11.3)	11	17	
Severe oesophagitis	29 (11.7)	15	14	
Severe gastritis	24 (9.7)	7	17	
Upper GI malignancy	13 (5.2)	6	7	
GAVE	11 (4.4)	0	11	
Polyps	8 (3.2)	6	2	
Oesophageal varices	5 (2)	1	4	
Dieulafoy lesion	5 (2)	5	0	
Duodenitis	1 (4)	0	1	
Endoscopic treatment				0.27
No treatment	185 (74.9)	73	112	
Adrenaline and clip	37 (15)	20	17	
Clip	12 (4.8)	6	6	
Haemospray	7 (2.8)	3	4	
Endoscopic management	4 (1.6)	0	4	
APC	1 (4)	0	1	
Variceal banding	1 (4)	0	1	
Immediate complications				0.175
None	212 (93.8)	95	117	
Bleeding	6 (2.7)	3	3	
Hypotension	5 (2)	0	5	
Death	2 (0.9)	1	1	
Abdominal symptoms	1 (0.4)	1	0	
Length of hospital stay				0.426
≤7 days	145 (58.5)	61	84	
>7 days	87 (35.1)	32	55	
90-day complications				0.516
Re-bleeding	8 (3.2)	0	8	
Pneumonia	8 (3.2)	1	7	
Re-admission in 90 days				0.998
None	152 (61.3)	69	83	
Yes	44 (17.7)	20	24	
30-day survival				0.484
Alive	203 (81.9)	91	112	
Dead	29 (11.7)	11	18	
90-day survival				0.180
Alive	184 (74.2)	85	99	
Dead	48 (19.4)	17	31	

Complications, length of stay, re-admission and survival

There were no immediate complications in 93.8% (n=212) of participants. Bleeding, hypotension and death occurred in 2.7% (n=6), 2% (n=5) and 0.9% (n=2) of participants, respectively. Hypotension did not occur in any patients aged 70-79 years and in five patients aged ≥80 years (0.03%, p=0.175). Most patients (n=145, 58.6%) had a length of stay of ≤7 days, and there was no statistically significant difference between groups (p=0.426).

In the ≥80 years group, eight patients had re-bled (0.05%) and seven had had pneumonia (0.05%), compared to zero and one patient in the 70-79 years group, respectively, but this was not found to be statistically significant (p=0.516). There was also no significant difference in re-admission rate within 90 days with 61.3% of patients not requiring re-admission (n=152, p=0.998).

Of patients aged 70-79 years and ≥80 years, 89.2% (n=91) and 76.6% (n=112) were alive at 30 days (p=4.84), respectively. At 90 days, 83.3% (n=85) of the younger cohort and 67.8% (n=99) of those ≥80 years were alive, a difference not found to be statistically significant (p=0.180) (Table 2).

## Discussion

In this study, we have evaluated the safety and efficacy of upper GI endoscopy in patients aged 70-79 years compared to those aged over 80 years presenting with acute upper GI bleeding.

There was no significant difference between the calculated Glasgow-Blatchford scores for the two groups, as both groups predominantly scored >0 and thus were deemed high risk (84% patients aged 70-79 years, n=86, and 78% aged over 80 years, n=114). We found that although most patients in both groups presented with melaena, a significant number of those ≥80 years presented with haematemesis (n=35, 24%, versus n=11, 10.8%, p=0.009). In contrast, other studies have found that younger patients are more likely to present with haematemesis, whereas more elderly patients presented with mild symptoms or subtle bleeding [9].

In both cohorts, it was most common to have normal findings at endoscopy; however, a statistically significant number of endoscopies in patients ≥80 years were reported as normal (n=47, 32%) than in the younger age group (n=27, 26%) (p=0.01). This is a similar finding to other studies, where 20%-34% of examinations did not reveal any abnormalities [11], and reinforces the importance of carefully selecting patients to undergo this procedure. Although peptic ulcer disease was the most common abnormal finding in both cohorts, it was noted that those aged over 80 years were more likely to have findings of gastric antral vascular ectasia (GAVE) (n=11 versus n=0) and oesophageal varices (n=4 versus n=1), as well as severe gastritis (n=17 versus n=7). In the literature, GAVE has been reported to account for 4% of non-variceal bleeding, which is in line with this study. It is known to affect elderly patients, particularly females, although one study found that hospitalisation with GAVE was most common in the 65-79 age group [12].

Most patients did not undergo endoscopic intervention (71.5% of 71-79-year-olds, n=73, and 76.7% of over 80s, n=112), and the most common intervention for both groups was injection of adrenaline and clip. Injection of adrenaline was found to be the most common intervention in other studies (78%-80% [9,13], with a lower proportion of patients requiring the application of two different haemostatic interventions (12% compared to 60% of endoscopic interventions in this study)) [13].

The majority of patients from both cohorts did not suffer any immediate complications (n=185, 93.8%). Although not found to be statistically significant, five patients from the ≥80 years group suffered from hypotension compared to none in the 70-79 years age group. Other studies have found that older patients are more likely to suffer from hypovolaemic shock following admission for UGIB, even in the absence of a significant fall in haemoglobin [13], confirming that elderly patients tolerate blood loss less well and develop haemodynamic instability earlier. This is important as hypovolaemic shock significantly increases the risk of death [9]. Another reason for this finding could be the use of sedative medications during endoscopy, as elderly patients are more sensitive to the adverse effects of sedatives [14]. It is also important to consider the pre-existing use of medications that may potentiate the hypotensive effect of sedatives, and elderly patients may be on multiple antihypertensive medications.

At 90 days, a greater proportion of patients ≥80 years were affected by complications when compared to the younger group, although not found to be statistically significant. Eight patients had suffered from re-bleeding, compared to none in the younger group (p=0.516). Previous studies have found that older patients found to have high-risk ulcers at endoscopy were more likely to suffer from re-bleeding than younger patients. It was suggested that this may be related to platelet dysfunction caused by the use of antiplatelets or non-steroidal anti-inflammatory drugs (NSAIDs) prior to admission, impairing haemostasis. They also noted increased late re-bleeding in elderly patients, possibly due to impaired healing of peptic ulcers [9]. More patients in the older group had developed pneumonia within 90 days (n=7 versus n=1). Despite this, we did not find a significant difference in the proportion of patients being re-admitted within this time frame, with 67.6% of the younger group (n=69) and 55.7% of the older group (n=83) not being re-admitted within 90 days. We also did not find a significant difference in the length of hospital stay between the two groups with 31% of 70-79-year-olds (n=32) and 38% of the ≥80-years-olds (n=55) staying more than seven days.

While the analysed data suggests that OGD remains a largely safe procedure in elderly patients presenting with an acute upper GI bleed, there is the suggestion, although not statistically significant, that older adults (≥80 years) may be more likely to suffer from complications including death. This was most noticeable when morbidity and mortality within the groups were compared at 90 days post-procedure. When assessing patient suitability for a procedure, it has been suggested that the use of frailty scoring such as the Rockwood Clinical Frailty Score may better inform the risk of poor outcomes than considering a patient’s age and co-morbidities in isolation [15]. Although open to some interobserver variability, especially among non-geriatricians, this scoring system can be easily applied following a brief discussion with the patient or their family members and could add useful information when deciding on when or if to proceed to OGD.

Limitations

In this study, we recorded whether a patient’s Glasgow-Blatchford was 0 or ≥1 but did not stratify patients further based on their score. It is accepted that a Glasgow-Blatchford score over zero suggests a high-risk bleed that is likely to require medical intervention, which includes transfusion, endoscopy or surgery. Established data also shows that a higher Glasgow-Blatchford score correlated with the likelihood that intervention is required, with scores greater than or equal to 6 being associated with a greater than 50% risk of needing intervention [16]. Data on indicators of haemodynamic instability at presentation and prior to the procedure was not collected, which would influence the risk of complications and poor prognostic outcomes.

This study did not evaluate the time to endoscopy in patients. This may be useful to establish whether prolonging the time before endoscopy is performed could allow for further optimisation of elderly, co-morbid patients and thus reduce the risk of complications.

Data was not collected on any non-invasive treatments initiated after endoscopy, such as the introduction of PPIs or rationalisation of antiplatelet or anticoagulant medications or NSAIDs. These measures, even in the absence of endoscopic therapy, may have influenced the risk of re-bleeding.

## Conclusions

In conclusion, we have found OGD to be a safe procedure in elderly adults presenting with acute upper gastrointestinal bleeding. However, a high proportion of emergency OGDs were reported as normal. This reinforces the importance of carefully selecting patients for this procedure, taking into account their individual risk factors and frailty and considering whether it may be more appropriate not to proceed to endoscopy or to delay the procedure to allow for medical optimisation. We recommend that further randomised controlled trials should be performed, recruiting large numbers of elderly patients, to compare outcomes in patients who receive medical management for UGIB and those who undergo OGD. We suggest that these trials could use clinical frailty scores to separate patients into mildly, moderately and severely frail cohorts. This would help produce guidelines on the management of frail, elderly patients presenting with UGIB.
